# Atherogenic Index of Plasma Predicts Futile Reperfusion and Early Deterioration After Successful Recanalization: A Multicenter Study of EVT-Treated LAA Stroke

**DOI:** 10.3390/biomedicines13092127

**Published:** 2025-08-31

**Authors:** Jong-Hee Sohn, Yong-Ho In, Chulho Kim, Joo Hye Sung, Minwoo Lee, Yerim Kim, Jae Jun Lee, Sang-Hwa Lee

**Affiliations:** 1 Department of Neurology, Chuncheon Sacred Heart Hospital, Hallym University College of Medicine, Chuncheon 24253, Republic of Korea; 2 Institute of New Frontier Research Team, Hallym University, Chuncheon 24253, Republic of Korea; inyh66@hallym.ac.kr (Y.-H.I.);; 3 Department of Neurology, Hallym Sacred Heart Hospital, Hallym University College of Medicine, Anyang 14068, Republic of Korea; 4 Department of Neurology, Kangdong Sacred Heart Hospital, Hallym University College of Medicine, Seoul 05355, Republic of Korea; 5 Department of Anesthesiology and Pain, Chuncheon Sacred Heart Hospital, Hallym University College of Medicine, Chuncheon 24253, Republic of Korea

**Keywords:** AIP, futile reperfusion, large vessel occlusion, endovascular treatment

## Abstract

**Background:** Futile reperfusion (FR), which is defined as successful revascularization without a favorable functional outcome, is a major limitation of endovascular treatment (EVT) for acute ischemic stroke. Although clinical and imaging predictors of FR have been studied, the role of systemic metabolic markers, such as the atherogenic index of plasma (AIP), remains unclear. No prior studies have examined the use of AIP in patients with large artery atherosclerosis (LAA)-related stroke. **Methods:** We analyzed data from four university-affiliated, prospectively maintained registries in the Republic of Korea (2015–2024). We included patients with anterior-circulation LVO who underwent EVT and achieved successful reperfusion. AIP was calculated as log(triglyceride/HDL-C) in mmol/L. The primary outcome was FR, defined as modified Rankin Scale (mRS) 3–6 at 3 months. The secondary outcome was early neurological deterioration (END). Multivariable logistic regression and ROC analysis were used. **Results:** Among the 406 LAA patients, 227 (55.9%) experienced FR, while 82 (20.2%) had END. Higher AIP quartiles were significantly associated with an increased risk of both FR and END (*p* for trend < 0.01). The highest AIP quartile (Q4 ≥ 0.26) had adjusted odds ratios of 4.34 (95% CI: 2.18–8.65) for FR and 9.62 (95% CI: 3.66–25.26) for END. The AUCs were 0.775 for FR and 0.726 for END. **Conclusions:** In a multicenter cohort of EVT-treated LAA stroke with successful reperfusion, elevated AIP independently predicted FR and END. AIP is a simple, widely available biomarker that may support pre-procedural risk stratification and inform post-reperfusion management after EVT.

## 1. Introduction

Endovascular treatment (EVT) has become a crucial option for treating acute ischemic stroke with large vessel occlusion (LVO) [1]. Despite advanced techniques and devices, up to 67% of LVO stroke patients experience futile reperfusion (FR), which is successful reperfusion without subsequent functional recovery (modified Rankin Scale [mRS] of 3 to 6 at three months) [2,3,4,5,6]. Predictors of FR include older age, higher initial stroke severity, poor collateral status, and reperfusion injury [7,8,9].

Several imaging or clinical predictors have been evaluated to improve FR after EVT [7,8,9,10,11]. The well-known imaging factor is collateral status, which plays a pivotal role in determining infarct growth and reperfusion outcomes; poor collaterals are consistently associated with FR [12,13,14]. Despite the emergence of these imaging predictors, the role of systemic metabolic biomarkers in predicting FR remains underexplored.

The atherogenic index of plasma (AIP) reflects a lipid profile associated with small dense low-density lipoprotein (LDL) particles and cardiovascular risk [15,16]. A higher AIP has been linked to poor outcomes in acute ischemic stroke, including increased early neurological deterioration (END) following EVT. It is particularly predictive of macrovascular atherosclerotic stroke subtypes [15,17]. However, its association with FR—especially in the context of LVO treated with EVT—has not been studied.

Since AIP reflects chronic atherosclerotic burden, systemic inflammation, and endothelial dysfunction [18,19], it is reasonable to assume that elevated AIP contributes to weaker collateral circulation, larger infarct cores, and impaired microvascular reperfusion. This can lead to FR despite successful clot removal [20]. Therefore, we hypothesize that higher pre-procedural AIP is associated with an increased risk of FR in LVO patients undergoing EVT.

We aimed to evaluate the impact of AIP on FR and stroke outcomes after EVT for LVO stroke with large artery atherosclerosis (LAA) using a multicenter registry database.

## 2. Methods

### 2.1. Study Population

We analyzed data from four university-affiliated, prospectively maintained multicenter stroke registries, enrolling consecutive patients with acute ischemic stroke between March 2015 and December 2024. From this cohort, we selected individuals who experienced LVO in the anterior circulation and underwent EVT. Only patients who achieved successful reperfusion, defined as a modified Thrombolysis in Cerebral Infarction (mTICI) score of 2b or 3, were included. We excluded patients based on the following criteria: (1) premorbid modified Rankin Scale (mRS) score > 2; (2) Alberta Stroke Program Early CT Score (ASPECTS) < 6; (3) time from symptom onset to groin puncture exceeding 12 h; (4) poor collateral circulation assessed by multiphasic CT angiography (mCTA); (5) prior use of statins before the index stroke; and (6) missing data on 3-month functional outcome.

### 2.2. Data Collection and Definition of Parameters

Demographic characteristics, clinical variables, laboratory findings, and stroke outcomes were extracted directly from a web-based, prospectively maintained registry. Initial stroke severity was assessed by trained neurologists using the National Institutes of Health Stroke Scale (NIHSS). Stroke etiology was determined using a modified version of the Trial of ORG 10,172 in Acute Stroke Treatment (TOAST) classification system [21]. Collateral circulation was graded as good, intermediate, or poor based on multiphasic CT angiography (mCTA), following the imaging criteria established by the Calgary Stroke Program [22]. Hyperlipidemia was defined as a physician diagnosis or the use of lipid-lowering therapy upon admission. Since this clinical definition reflects prior diagnoses and treatments, there may not be a monotonic relationship with baseline AIP.

Upon admission, patients underwent standardized laboratory testing, including complete blood counts, fasting glucose, high-sensitivity C-reactive protein, renal function tests, and lipid profiles. The AIP was calculated using the base-logarithm of the triglyceride-to-HDL cholesterol ratio with values expressed in mmol/L [23]. This ratio was obtained from the first blood draw, which was taken upon hospital arrival and preferably before EVT.

The primary outcome was FR, defined as an mRS score of 3 to 6 at 3 months despite achieving successful reperfusion (mTICI grade 2b–3) [24]. To ensure consistency in mTICI grading, angiographic images were independently reviewed by two experienced stroke neurologists (M. L. and Y. K.) who were blinded to clinical outcomes; interrater agreement was excellent (intraclass correlation coefficient [ICC], 0.90; *p* < 0.001). The secondary outcome was early neurological deterioration (END), defined as a decline of more than 2 points in the total NIHSS score or a worsening of at least 1 point in motor function within 72 h after admission, relative to the initial NIHSS score. END events were predefined and adjudicated into three etiologies; (1) END–prog, infarct extension within the index vascular territory after EVT without imaging evidence of new-territory ischemia or symptomatic hemorrhagic transformation; (2) END–rec, a new ischemic event in a distinct vascular territory or new lesion(s) explaining the deterioration; and (3) symptomatic hemorrhagic transformation (SHT), neurological worsening accompanied by hemorrhagic transformation on imaging [25]. Two expert vascular neurologists (M. Lee and S.-H. Lee) performed etiological classification in a blinded manner, yielding high interrater reliability (ICC, 0.89; *p* < 0.001).

### 2.3. Statistical Analysis

We hypothesized that an elevated AIP would be associated with a higher risk of FR after EVT. Continuous variables were summarized as means with standard deviations (SDs), and categorical variables were presented as counts and their corresponding percentages. We analyzed baseline characteristics across increasing quartiles of AIP. Group comparisons of normally distributed continuous variables were assessed using one-way ANOVA, and group comparisons of non-normally distributed variables were evaluated using the Kruskal–Wallis test. Differences in categorical variables were tested using Pearson’s chi-square test or Fisher’s exact test, as appropriate. We initially treated AIP as a continuous variable and subsequently categorized it into quartiles, with the first quartile serving as the reference group. Multivariable logistic regression was used to estimate the independent association between AIP quartiles and stroke outcomes while adjusting for potential confounders. Covariates were selected based on univariate *p*-values less than 0.1 and clinical relevance. Odds ratios (ORs) with 95% confidence intervals (CIs) were calculated for both crude and adjusted models.

As a sensitivity analysis, we derived a composite index AIP-BMI (AIP × BMI) [26]. We modeled AIP-BMI in quartiles (Q1 reference) and estimated adjusted ORs for FR and END using logistic regression analysis. Additionally, we conducted pre-specified subgroup analyses of FR according to age (≥60 vs. <60 years), diabetes status, and LDL-C (<3.4 vs. ≥3.4 mmol/L). We also fit multiplicative interaction terms (AIP quartile × modifier) and reported the likelihood ratio and joint Wald *p*-value.

To explore potential nonlinear associations between AIP and FR, we performed restricted cubic spline regression, adjusting for the same covariates used in the logistic model. Additionally, we derived optimal AIP cutoff values for predicting FR and END using a receiver operating characteristic (ROC) curve based on the multivariate model. We conducted all analyses using SPSS version 21.0 (IBM Corp., Armonk, NY, USA) and R software version 4.0.3 (R Foundation for Statistical Computing, Vienna, Austria).

## 3. Results

**Figure 1:**
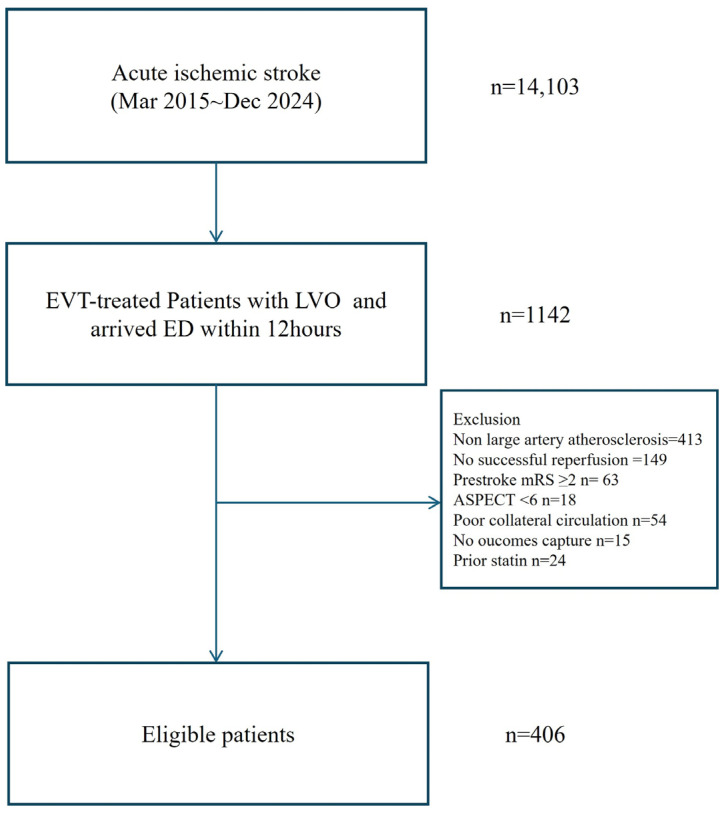
Flow chart of this study. Abbreviation: EVT, endovascular treatment; LVO, large vessel occlusion; ED, emergency department; mRS, modified Rankin Scale; ASPECTS, Alberta Stroke Program Early CT Score.

Of the 14,103 consecutive patients with acute ischemic stroke, 1142 (8.1%) underwent EVT for anterior circulation LVO. After applying the study’s eligibility criteria, 406 patients were included in the final analysis (Figure 1). Participants were stratified into four groups based on their baseline AIP quartile: the first quartile (Q1, ≤−0.1), the second quartile (Q2, −0.13 to 0.02), the third quartile (Q3, 0.02 to <0.26), and the fourth quartile (Q4, ≥0.26). Baseline demographic, clinical, and laboratory characteristics were similar across groups, though higher AIP quartiles were associated with a greater prevalence of current smoking, elevated HbA1c levels, and an increased LDL cholesterol trend (Table 1).

**Figure 2:**
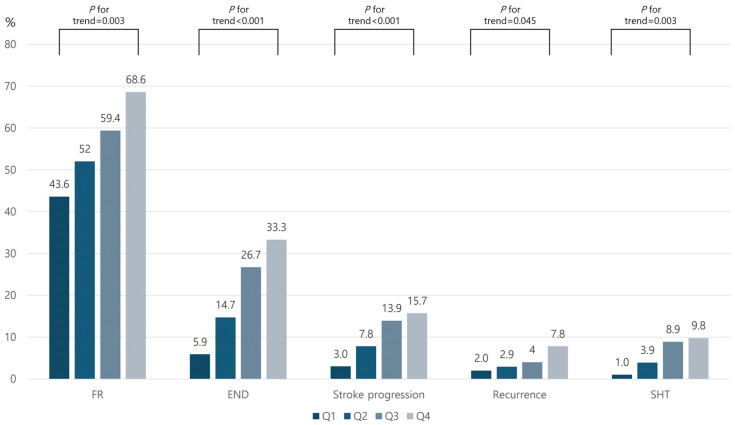
Comparison of stroke outcomes between quartiles of AIP level. Abbreviation: AIP, Atherogenic Index of Plasma; FR, futile reperfusion; END, early neurologic deterioration; SHT, symptomatic hemorrhagic transformation; Q1, first quartile; Q2, second quartile; Q3, third quartile; Q4, fourth quartile.

Of the 406 enrolled patients, 227 (55.9%) experienced FR and 82 (20.2%) developed END during hospitalization. The frequency of both FR and END increased progressively with higher AIP quartiles (*p* for trend = 0.003 for FR; *p* < 0.001 for END). When END was categorized by etiology, the rates of stroke progression, recurrence, and SHT were all higher in the highest AIP quartile (Figure 2).

**Figure 3:**
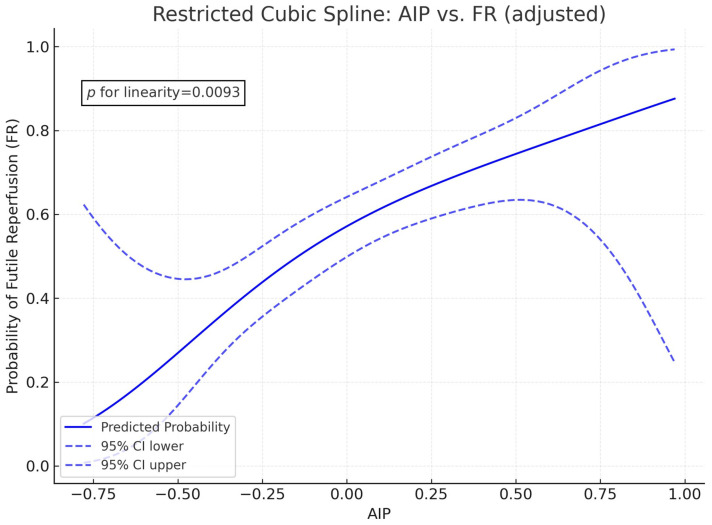
Restricted cubic spline showing correlation between AIP levels and FR. Abbreviation: AIP, Atherogenic Index of Plasma; FR, futile reperfusion. Adjusted for age, male sex, initial NIHSS, time interval from onset to arrival, history of HTN, smoking, LDL level, and glycated hemoglobin.

Multivariable logistic regression analysis revealed that patients in higher AIP quartiles were at a significantly greater risk of FR and END (Table 2 and Supplementary Table S1). Regarding the etiologies of END, a higher AIP was associated with stroke progression and SHT, but not with stroke recurrence (Table 2 and Supplementary Table S2). Furthermore, restricted cubic spline analysis revealed a linear, dose-dependent association between AIP and FR risk (*p* for linearity = 0.0093; Figure 3).

**Figure 4:**
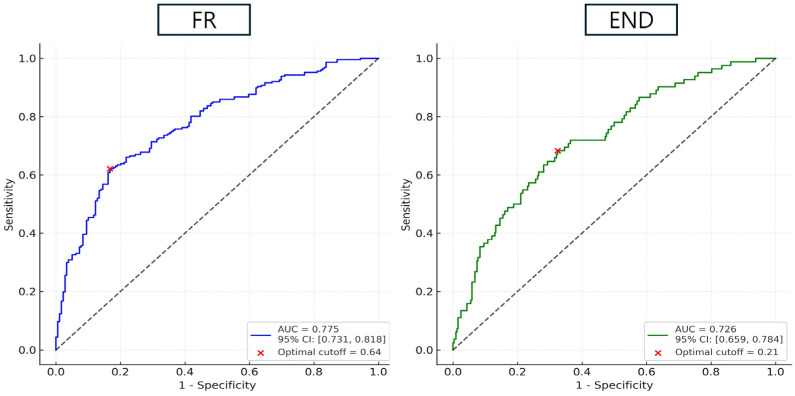
ROC curve showing predictive ability of AIP for FR and END. Abbreviation: ROC, receiver operating characteristic; AIP, Atherogenic Index of Plasma; FR, futile reperfusion; END, early neurologic deterioration.

ROC analysis demonstrated the AIP’s good predictive accuracy for both outcomes: the area under the curve (AUC) was 0.775 [95% CI: 0.731–0.818] for FR and 0.726 [95% CI: 0.659–0.784] for END (both *p* < 0.001). The optimal AIP cutoff values were 0.64 for predicting FR and 0.21 for predicting END (Figure 4).

In a sensitivity analysis, higher AIP-BMI quartiles were associated with an increased risk of FR (Q3: OR 2.69 [1.40–5.15], *p* = 0.003; Q4: OR 3.26 [1.64–6.47], *p* = 0.001) and END (Q3: OR 6.20 [2.40–16.06], *p* < 0.001; Q4: OR 7.17 [2.74–18.76], *p* < 0.001) compared to Q1. Full estimates are provided in Supplementary Table S3. A subgroup analysis (Supplementary Table S4) revealed that, among patients aged 60 years and older, the adjusted OR were 3.14 (95% CI: 1.46–6.76) for Q3 and 5.21 (95% CI: 2.33–11.67) for Q4 (both *p* ≤ 0.003). Among non-diabetic patients, the ORs were 2.39 (1.13–5.05) and 3.70 (1.65–8.28) for Q3 and Q4, respectively. Among diabetic patients, the OR for Q4 was 7.38 (1.66–32.82), *p* = 0.009. In the LDL-C < 3.4 mmol/L stratum, the ORs were 2.50 (1.22–5.12) and 5.48 (2.52–11.92) for Q3 and Q4, respectively (both *p* ≤ 0.012). Estimates in the LDL-C ≥ 3.4 mmol/L stratum were directionally positive but imprecise. Interaction tests (AIP quartile × age/diabetes/LDL) were non-significant (all *p* > 0.20).

## 4. Discussion

The main findings of this study are as follows: (1) Elevated AIP was significantly associated with both FR and END in LAA-related stroke after EVT; and (2) AIP reliably predicted FR and END after EVT. Together, these results imply that AIP, a surrogate marker of atherogenic dyslipidemia, could be a new predictor of unfavorable outcomes despite successful reperfusion in LAA stroke.

While FR remains a significant limitation of EVT for acute ischemic stroke, previous studies have primarily focused on imaging biomarkers, such as infarct core size, collateral status, and procedural time metrics, as predictors of outcome. In contrast, our study emphasizes the prognostic relevance of systemic metabolic health, particularly as reflected by the AIP. An elevated AIP indicates a dyslipidemic state characterized by high triglyceride and low HDL-C levels. These levels suggest an increase in atherogenic remnant particles, small dense LDL, and reduced reverse cholesterol transport. These lipid abnormalities are strongly associated with endothelial dysfunction, which is characterized by impaired nitric oxide (NO) production, increased oxidative stress, and vascular inflammation [27,28,29]. At the cerebrovascular level, chronic endothelial injury can hinder the development of pial and leptomeningeal collateral networks, thereby reducing baseline perfusion buffering capacity during arterial occlusion [30]. Furthermore, elevated AIP is associated with an increased systemic inflammatory burden. This is characterized by higher levels of circulating cytokines (e.g., IL-6 and TNF-α) and enhanced oxidative damage. Both of these factors upregulate matrix metalloproteinases (MMPs) and promote blood–brain barrier (BBB) breakdown [31]. This proinflammatory environment may facilitate no-reflow phenomena. These phenomena involve microvascular obstruction, leukocyte plugging, and pericyte-mediated vasoconstriction, which prevent adequate tissue reperfusion even after macrovascular recanalization [32]. Despite technically successful EVT, such microcirculatory failure contributes to infarct core expansion. Therefore, elevated AIP may indicate impaired macrovascular collateralization and deficient microvascular perfusion. This offers a plausible explanation for its strong association with FR in our LAA stroke cohort.

Our restricted cubic spline analysis revealed a significant and approximately linear association between AIP and the risk of FR, suggesting that even modest elevations in atherogenic lipid profiles may incrementally increase the likelihood of suboptimal outcomes after EVT. This finding supports the notion that lipid dysregulation exerts a dose-dependent deleterious effect on cerebral perfusion dynamics, likely through a combination of impaired collateral flow, microvascular dysfunction, and inflammation-mediated no-reflow phenomena [20,33]. Furthermore, our multivariable prediction model results using the ROC are notable, as many previous EVT outcome prediction tools primarily rely on imaging features, procedural metrics, or baseline stroke severity. Including AIP, a routinely available and cost-effective laboratory marker, in these models may enable real-time, preprocedural risk stratification in acute stroke care settings [34,35]. In addition, identifying patients with high AIP levels could facilitate targeted prevention strategies, including the early initiation of lipid-lowering therapies with pleiotropic, endothelial-protective effects (e.g., statins or PCSK9 inhibitors) [36]. Such strategies may be particularly relevant for patients with LAA stroke, as dyslipidemia is a key modifiable risk factor for this condition.

Similarly to previous studies, our study also suggests that AIP is associated with END [15,37]. Interestingly, among the etiologies of END, our study identified stroke progression and SHT as the dominant causes in patients with elevated AIP. These results imply that AIP-related pathophysiology reflects not only systemic vascular risk but also plays a mechanistic role in clinical instability after recanalization. While FR primarily indicates microvascular reperfusion failure despite recanalization, END emphasizes the clinical manifestation of downstream injury progression in the hours to days following EVT. In cases of stroke progression, elevated AIP may contribute to ongoing ischemic damage by exacerbating impaired autoregulation, reducing capillary-level perfusion, and sustaining inflammatory activation in the penumbra. These conditions, influenced by endothelial dysfunction and oxidative stress, can lead to expansion of the infarct core even after technically successful reperfusion [17,29]. Interestingly, our subgroup analysis of END etiologies revealed that AIP was not significantly associated with stroke recurrence during the early post-EVT period. This suggests that, although AIP reflects systemic atherogenic burden and endothelial dysfunction, it may not directly mediate the acute thromboembolic mechanisms responsible for early stroke recurrence. Stroke recurrence within 72 h is often driven by underlying cardioembolic sources, procedural embolization, or residual unstable atherosclerotic plaques. These factors may not be fully captured by static lipid indices, such as AIP [38]. Moreover, patients with LAA stroke in our cohort underwent successful mechanical reperfusion, which may have minimized the likelihood of early recurrent large-vessel thrombotic events. AIP may serve as both a static risk indicator and a pathophysiologic link between lipid metabolism, microvascular injury, and clinical outcomes after large vessel recanalization. Incorporating AIP into risk stratification frameworks could help identify patients at increased risk for ischemic progression or hemorrhagic conversion, enabling personalized post-reperfusion management.

Notably, our data indicate that patients with higher AIP may be more susceptible to SHT after successful reperfusion. Previous reports on lipids and small vessel disease (SVD) imaging markers are heterogeneous. Several studies describe an inverse relationship between dyslipidemia and cerebral microbleeds. Very low LDL-C has been associated with a higher risk of intracerebral hemorrhage or microbleeds, which underscores the complexity of chronic cerebral SVD biology [39,40,41]. In contrast, our cohort focuses on post-recanalization outcomes, in which microvascular no-reflow, endothelial/pericyte dysfunction, and BBB disruption dominate tissue fate despite macrovascular success [42]. In this acute reperfusion environment, AIP can be considered an integrator of proatherogenic dyslipidemia and low-grade inflammation, which are linked to endothelial dysfunction, rather than a direct driver of a single molecular pathway [26]. The inflammatory and oxidative environment associated with atherogenic dyslipidemia can increase MMP expression, which may destabilize the BBB and facilitate hemorrhagic conversion after reperfusion [31,43]. However, our data does not establish a direct causal relationship between AIP and MMP-9. This framework bridges the gap between the seemingly protective lipid signals observed in chronic SVD and the detrimental effects of metabolic-inflammatory dysregulation during acute reperfusion.

Our threshold estimates naturally diverge from previously reported AIP cutoffs (e.g., 0.112) due to substantial differences in the study population, endpoints, and analytic scale. We restricted the cohort to EVT-treated LAA strokes with successful reperfusion and excluded pre-stroke statin users. This narrowed the case mix to a metabolically and angiographically distinct subgroup prone to FR/END dynamics. Furthermore, our thresholds were obtained from multivariable model probabilities. In contrast, many prior studies used AIP as a single continuous predictor against disability outcomes in heterogeneous AIS populations.

One of the major strengths of our study is our homogeneous patient population. We only included individuals who had an LAA-related stroke and good collateral status. This minimizes the influence of confounding factors, such as cardioembolic or cryptogenic etiologies, which may have distinct vascular and metabolic profiles. Additionally, we excluded patients with a history of statin use because statins can significantly alter lipid parameters and lower AIP values. Thus, we reduced the potential for treatment-induced bias and ensured that the baseline AIP more accurately reflected the endogenous atherogenic burden at the time of stroke onset. Consequently, our findings regarding AIP are more applicable to real-world EVT pathways that use similar selection criteria and post-procedural care. Our findings also provide a coherent biological framework for incorporating a simple metabolic biomarker into risk stratification after successful recanalization. Nonetheless, this study has several limitations. First, the observational design precludes any causal inference between elevated AIP and FR or END. Second, although AIP was derived from standardized fasting lipid panels, acute-phase changes during the early post-stroke period may have influenced triglyceride and HDL-C levels. In addition, post-treatment lipid profiles were not consistently collected across participating centers, so longitudinal changes in AIP after EVT or statin initiation could not be evaluated. Third, although we adjusted for multiple covariates, unmeasured confounders such as inflammatory biomarkers or genetic predispositions could have influenced the outcomes. Fourth, we did not calculate the final infarct volume after EVT to support our results. Lastly, the study population was derived from a Korean multicenter registry, which limits the generalizability of the results to other ethnic groups.

In conclusion, AIP is an independent predictor of FR and early deterioration after EVT in patients with LAA stroke. These findings suggest the potential role of systemic metabolic biomarkers in stratifying EVT candidates and guiding adjunctive strategies to improve microvascular reperfusion and clinical recovery.

## Figures and Tables

**Table 1:** Baseline characteristics according to AIP quartiles.

	Q1 (*n* = 101)	Q2 (*n* = 102)	Q3 (*n* = 101)	Q4 (*n* = 102)	*p*-Value
Age, year (SD)	69.2 (1.4)	69.7 (12.9)	66.2 (14.4)	66.6 (12.7)	0.15
Male, % (SD)	61 (60.4)	62 (60.8)	67 (66.3)	58 (56.9)	0.58
BMI, kg/cm^2^ (IQR)	23 (21–25)	23 (21–25)	23 (22–26)	23 (22–26)	0.14
Initial NIHSS, score (IQR)	14 (9–18)	14 (10–17)	14 (9–18)	13 (8–17)	0.28
Time Interval from stroke onset to arrival, h (IQR)	1.38 (0.73–3.97)	1.29 (0.63–4.06)	1.92 (0.96–4.87)	1.46 (0.67–3.70)	0.08
Time Interval from arrival to groin puncture, min (IQR)	116 (90–144)	105 (76–141)	104 (77–147)	106 (81–153)	0.64
Prior stroke, % (SD)	20 (19.8)	26 (25.5)	22 (21.8)	17 (16.7)	0.48
Hypertension, % (SD)	61 (60.4)	61 (59.8)	54 (53.5)	61 (59.8)	0.72
Diabetes mellitus, % (SD)	22 (21.8)	22 (21.6)	28 (27.7)	36 (35.3)	0.09
Hyperlipidemia, % (SD)	26 (25.7)	17 (16.7)	19 (18.8)	22 (21.6)	0.42
Current smoking, % (SD)	9 (8.9)	3 (2.9)	14 (13.9)	22 (21.6)	<0.001
Prior antithrombotics, % (SD)	18 (17.8)	25 (24.5)	25 (24.8)	25 (24.5)	0.58
Reperfusion therapy, % (SD)					0.14
EVT only	60 (59.4)	44 (43.1)	51 (50.5)	50 (49.0)	
IVT + EVT	41 (40.6)	58 (56.9)	50 (49.5)	52 (51.0)	
ASPECTS, score (IQR)	8 (7–9)	8 (7–9)	8 (7–9)	8 (7–9)	
Collateral status, % (SD)					0.75
Good	58 (57.4)	61 (59.8)	60 (59.4)	66 (64.70)	
Intermediate	43 (42.6)	41 (4.02)	41 (40.6)	36 (35.3)	
Medications at discharge					
Antiplatelet agents	92 (91.1)	84 (82.4)	82 (81.2)	83 (81.5)	0.17
Antihypertensive agents	89 (88.1)	85 (83.3)	82 (81.2)	80 (78.4)	0.31
Antidiabetic agents	50 (49.5)	44 (43.1)	46 (45.5)	47 (46.1)	0.85
Statin	84 (83.2)	78 (76.5)	74 (73.3)	81 (79.4)	0.37
Platelet count, ×1000/μL (SD)	214 (23.1)	148 (26.6)	148 (24.0)	148 (25.6)	0.18
Creatinine, mg/dL (SD)	1.08 (0.69)	1.18 (1.64)	1.03 (0.29)	1.11 (0.81)	0.76
LDL, mmol (SD)	2.2 (0.8)	2.6 (0.9)	2.8 (0.9)	2.7 (1.1)	<0.001
Glycated hemoglobin, % (SD)	5.9 (1.1)	5.9 (0.9)	6.3 (1.4)	6.5 (1.6)	0.002
CRP, mg/dL (SD)	9.4 (16.9)	9.9 (12.9)	13.4 (28.6)	16.1 (31.1)	0.15
Initial glucose, mg/dL (SD)	143 (62.1)	139 (51.9)	154 (66.3)	154 (66.3)	0.32
SBP, mmHg (SD)	144 (23.1)	148 (26.6)	148 (25.6)	147 (24.8)	0.47

Abbreviation: Q1, first quartile; Q2, second quartile; Q3, third quartile; Q4, forth quartile; SD, standard deviation; IQR, interquartile range; BMI, body mass index; NIHSS, National Institute Health Stroke scale; EVT, endovascular treatment; IVT, intravenous thrombolysis; ASPECTS, Alberta Stroke Program Early CT Score; LDL, low density lipoprotein; CRP, C-reactive protein; SBP, systolic blood pressure.

**Table 2:** Multivariate analysis showing the impact of quartiles of AIP on FR and END after EVT.

	FR		END		Stroke Progression		Stroke Recurrence		SHT	
	OR *	95% CI	OR *	95% CI	OR *	95% CI	OR *	95% CI	OR *	95% CI
AIP in Quartiles										
Q1	reference		reference		reference		reference		reference	
Q2	1.48	0.80–2.74	2.96	1.08–8.11	2.78	0.71–10.92	1.52	0.23–10.09	4.87	0.52–37.45
Q3	2.74	1.43–5.26	7.25	2.75–19.14	5.92	1.61–21.87	1.38	0.22–8.78	16.01	1.90–134.96
Q4	4.31	2.16–8.61	9.08	3.48–23.67	6.34	1.73–23.27	4.62	0.80–25.02	11.87	1.43–98.20

Abbreviation: AIP, Atherogenic Index of Plasma; FR, futile reperfusion; END, early neurologic deterioration; SHT, symptomatic hemorrhagic transformation; OR, odds ratio; CI, confidence interval; Q1, first quartile; Q2, second quartile; Q3, third quartile; Q4, forth quartile. * Adjusted for age, male sex, initial NIHSS, time interval from onset to arrival, history of HTN, smoking, LDL level, glycated hemoglobin.

## Data Availability

The datasets generated during and/or analysed during the current study are available from the corresponding author on reasonable request.

## Supplementary Materials

The following supporting information can be downloaded at: https://www.mdpi.com/article/10.3390/biomedicines13092127/s1, Table S1: Multivariate analysis showing impact of AIP on FR and END after successful EVT. Table S2: Multivariate analysis showing impact of AIP on each etiology of END after successful EVT. Table S3: Logistic regression analysis showing the impact of AIP-BMI quartiles on FR and END. Table S4: Subgroup analysis showing impact of AIP quartiles on FR by age, diabetes, and LDL strata and interaction effect.
