# Supplementary material for: Atherogenic Index of Plasma Predicts Futile Reperfusion and Early Deterioration After Successful Recanalization: A Multicenter Study of EVT-Treated LAA Stroke
Source: Biomedicines. 2025 Aug 31;13(9):2127. doi: 10.3390/biomedicines13092127 (PMC12467022; doi:10.3390/biomedicines13092127)
Supplement: Supplementary file 1 [file biomedicines-13-02127-s001.zip › biomedicines-3834276-supplementary.pdf]

Supplementary Table S1. Multivariate analysis showing impact of AIP on FR and END after successful EVT.

	FR			END		
	OR	95%CI	*p*-value	OR	95%CI	*p*-value
AIP quartiles						
Q1	reference			reference		
Q2	1.48	0.80–2.74	0.22	2.96	1.08–8.11	0.04
Q3	2.74	1.43–5.26	0.002	7.25	2.75–19.14	< 0.001
Q4	4.31	2.16–8.61	< 0.001	9.08	3.48–23.67	< 0.001
Age	1.04	1.02–1.06	< 0.001	1.02	0.995–1.04	0.12
Male	0.89	0.36–0.95	0.03	0.78	0.45–1.36	0.38
Time interval from Stroke onset to arrival	1.02	0.99–1.05	0.18	0.99	0.95–1.03	0.48
Initial NIHSS	1.11	1.07–1.16	< 0.001	0.99	0.95–1.04	0.62
HTN	1.37	0.84–2.22	0.21	0.97	0.28–1.85	0.49
smoking	0.86	0.41–1.79	0.69	0.72	0.28–1.85	0.49
LDL	0.99	0.78–1.27	0.95	0.78	0.59–1.03	0.08
Hba1c	1.3	1.07–1.57	0.01	1.19	0.999–1.43	0.07

Supplementary Table S2. Multivariate analysis showing impact of AIP on each etiology of END after successful EVT.

	Stroke progression			Stroke recurrence			SHT		
	OR	95%CI	*p*-value	OR	95%CI	*p*-value	OR	95%CI	*p*-value
AIP quartiles									
Q1	reference			reference			reference		
Q2	2.78	0.71–10.92	0.14	1.52	0.23–10.09	0.66	4.87	0.52–45.37	0.16
Q3	5.92	1.61–21.87	0.01	1.38	0.22–8.78	0.74	16.01	1.90–134.96	0.01
Q4	6.34	1.73–23.27	0.01	4.62	0.8–25.02	0.08	11.87	1.43–98.20	0.02
Age	1.01	0.98–1.05	0.38	1.05	0998–1.11	0.06	1.003	0.96–1.04	0.88
Male	0.89	0.44–1.82	0.76	1.15	0.38–3.50	0.81	0.57	0.22–1.46	0.24
Time interval from Stroke onset to arrival	0.97	0.91–1.03	0.32	1.04	0.99–1.09	0.12	0.87	0.72–1.05	0.15
Initial NIHSS	0.99	0.93–1.04	0.61	0.94	0.86–1.03	0.18	1.03	0.96–1.11	0.43
HTN	1.25	0.59–2.67	0.56	0.26	0.08–0.85	0.03	1.81	0.63–5.18	0.27
Smoking	1.02	0.33–3.13	0.97	0	0	0.997	1.12	0.21–5.85	0.90
LDL	0.95	0.68–1.35	0.79	1.12	0.663–1.88	0.68	0.53	0.31–0.88	0.01
Hba1c	0.95	0.73–1.23	0.70	1.49	1.11–1.997	0.01	1.20	0.91–1.58	0.21

Supplementary Table S3. Logistic regression analysis showing the impact of AIP-BMI quartiles on FR and END

	FR			END		
AIP-BMI in quartiles	OR*	95%CI	*p*-value	OR*	95%CI	*p*-value
Q1	reference			reference		
Q2	1.30	0.69–2.41	0.42	2.11	0.75–5.92	0.16
Q3	2.69	1.40–5.15	0.003	6.2	2.40–16.06	< 0.001
Q4	3.26	1.64–6.47	0.001	7.17	2.74–18.76	< 0.001

*Adjusted for age, male sex, initial NIHSS, HTN, HbA1c

Supplementary Table S4. Subgroup analysis showing impact of AIP quartiles on FR by age, diabetes, and LDL strata and interaction effect.

	AIP Q1	AIP Q2	AIP Q3	AIP Q4	*p* for interaction
Age					
< 60	ref	0.60 (0.15–2.97)	1.49 (0.34–5.97)	3.0 (0.67–13.35)	0.70
≥ 60	ref	1.83 (0.93–3.63)	3.14 (1.46–6.76)	5.21 (2.33–11.67)	
Diabetes					
No	ref	1.86 (0.92–3.78)	2.39 (0.92–3.78)	3.70 (0.65–8.28)	0.23
Yes	ref	0.77 (0.19–3.04)	4.10 (0.89–18.84)	7.38 (1.66–32.82)	
LDL-C					
< 3.4 mmol/L	ref	1.49 (0.77–2.90)	2.50 (1.22–11.92)	5.48 (2.52–11.92)	0.22
≥ 3.4 mmol/L	ref	2.01 (0.27–15.12)	4.27 (0.58–31.71)	2.31 (0.31–17.30)	
